# Vitreous levels of Lipocalin-2 on patients with primary rhegmatogenous retinal detachment

**DOI:** 10.1371/journal.pone.0227266

**Published:** 2019-12-31

**Authors:** Georgios Batsos, Eleni Christodoulou, Georgios Vartholomatos, Petros Galanis, Maria Stefaniotou

**Affiliations:** 1 Ophthalmology Department, University Hospital Of Ioannina, Ioannina, Greece; 2 Haematology Laboratory Unit of Molecular Biology, University Hospital Of Ioannina, Ioannina, Greece; 3 Center for Health Services Management and Evaluation, National and Kapodistrian University of Athens (NKUA), Athens, Greece; Massachusetts Eye & Ear Infirmary, Harvard Medical School, UNITED STATES

## Abstract

**Purpose:**

To measure vitreous levels of Lipocalin2 (LCN2) in patients with rhegmatogenous retinal detachment (RRD) and investigate potential association with presence of proliferative vitreoretinopathy (PVR).

**Materials and methods:**

Collection of undiluted vitreous samples from 24 patients suffering from RRD and 10 control patients undergoing vitrectomy for: vitreomacular traction (VMT) (n = 2), idiopathic epiretinal membrane (iERM) (n = 6) and full thickness macular hole (FTMH) (n = 2). Quantitative analysis of LCN2 has been made with flow cytometry. Lens status, duration of symptoms, quadrants of detachment, as well as level of PVR, were assessed. Statistical analysis included Mann-Whitney test, Kruskal-Wallis test, t-test, Spearman’s correlation coefficient and Fisher's exact test.

**Results:**

Median LCN2 was significantly higher in the RRD group as compared to control (p<0.001). Within the RRD group there was a positive correlation between LCN2 and PVR grade (r_s_ = 0.94, p<0.001). Median LCN2 was 35,759 pg/ml (IR = 55,347) in grade C PVR, 9,387 pg/ml (IR = 3721) in grade B, 4,917 pg/ml (IR = non computable) in grade A and 3,921 pg/ml (2132) in the no PVR group. Median LCN2 was also significantly higher in pseudophakic patients as compared to phakic patients (p = 0.007). LCN2 also correlates with the extend of detachment (≤2 vs >2 quadrants, p<0.001) as well as with duration of symptoms (r_s_ = 0.87, p<0.001). After multivariate linear regression analysis, only PVR was independently related with LCN2 concentration. In particular, increased PVR grading was associated with increased LCN2 concentration (coefficient b = 2.97, 95% confidence interval = 1.89 to 4.67, p<0.001).

**Conclusion:**

A positive correlation between vitreous levels of LCN2 and PVR grading reveals a potential role in the pathogenesis and progression of PVR. Further studies could elucidate if LCN2 could be a therapeutic target.

## Introduction

Nowadays, single operation success rate in the management of retinal detachment is approximately 80% [1–4]. However, nearly 10% of patients will need repeat surgery due to the risk of recurrence [5] mostly from Proliferative Vitreoretinopathy (PVR), which remains an unsolved problem for patients and vitreoretinal surgeons.

PVR is characterized by cell migration and proliferation in the peri-retinal space accompanied by the development of pre-retinal and sub-retinal membranes. As a result of the aforementioned as well as additional alterations [6, 7], retina traction with subsequent contraction ensue. It occurs in 5–10% of rhegmatogenous retina detachment (RRD) cases, and in 75% of re-detachments after a successful surgical repair [8].

Many factors and mediators are implicated in PVR pathogenesis [7, 9–14]. Various studies and clinical trials for the pharmaceutical treatment or prevention of PVR have been performed [15]; yet the standard treatment remains surgical [16].

Latest research is focused on determining biomarkers related to PVR [7, 17–19]. One of the major contributors in PVR development is inflammation [8, 12, 20, 21]. The inflammatory process which is initiated after a rhegmatogenous retinal detachment, is driven by various cytokines and growth factors [15]. Moreover the degree of inflammation is related to the remodeling mechanisms leading to PVR formation [7]. Lipocalin-2 (LCN2), also known as neutrophil gelatinase-associated lipocalin (NGAL), is an adipokine involved in a variety of processes such as metabolic homeostasis, apoptosis, infection, immune response, or inflammation. Elevated levels of lipocalin2 have also been measured in the aqueous humor of patients with idiopathic acute anterior uveitis (AAU) [22] as well as in patients with branch retinal vein occlusion (BRVO) [23]. LCN2 levels have been proposed as a biomarker for various inflammatory diseases [24, 25], [26], [27], [28] [29], [30] [31–33] but its role in RRD and PVR has not been examined thus far and is the aim of this study.

## Materials and methods

This study was conducted at the University Hospital of Ioannina, Greece, between March and July 2019, after receiving approval from the hospital’s ethics committee “Scientific Board” (March 2019). During that period, patients recruited from the hospital’s Ophthalmology clinic (Vitreoretinal Department). Written informed consent was obtained before each surgery. The study is adherent to the tenets of the Declaration of Helsinki.

Vitreous samples were collected from 24 RRD patients (17 males, 7 females) aged between 35 and 86 years. Our control group comprised of 10 patients (3 males and 7 females), undergoing vitrectomy for various vitreoretinal pathologies other than RRD; 6 were diagnosed with idiopathic epiretinal membrane (iERM), 2 with vitreomacular traction syndrome (VMT), and 2 with full-thickness macular hole (FTMH). Age in the control group ranged from 51 to 84 years. Complete ophthalmic examination was performed to all patients. In the RRD group, we also assessed lens status (2 groups: phakic, pseudophakic), quadrants of involvement (2 groups: 1–2, 3–4), symptom duration (3 groups <10, 10–30, >30 days), and PVR grading (if present) according to the Retina Society Classification (1991) [34–36].

Patients with a history of ocular trauma, prior ophthalmic surgery other than phacoemulsification, history of diabetes mellitus, or an undergoing systemic inflammatory disease were excluded from this study.

All patients were operated by the same surgeon with standard 25G pars plana vitrectomy. Approximately 0.5ml of undiluted vitreous core sample was collected at the beginning of vitrectomy, just before the opening of the infusion cannula. Samples were stored at -80 °C.

### Flow cytometry

**Fig 1:**
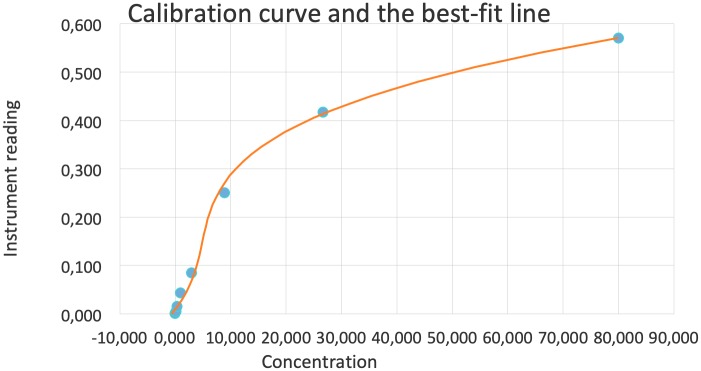
Standard curve. The horizontal axis represents the concentration of NGAL in pg/mL and the vertical axis the mean fluorescence intensity (MFI).

Results were analyzed via the CellQuest software (Becton-Dickinson). Sample analysis was performed using AimPlex multiplex assay on a FACSCalibur (Becton-Dickinson) flow cytometer via a three-step process. A 60-minute incubation with an antibody-conjugated bead was followed by a 30-minute biotinylated detection step; the latter preceded a final 20-minute Streptavidin-Phycoerythrine (PE) incubation. LCN2 (NGAL) concentrations were obtained by comparing the fluorescent signals to those of a standard curve (Fig 1). Results were calculated from such standard curves and expressed as picograms per milliliter (pg/mL).

**Fig 2:**
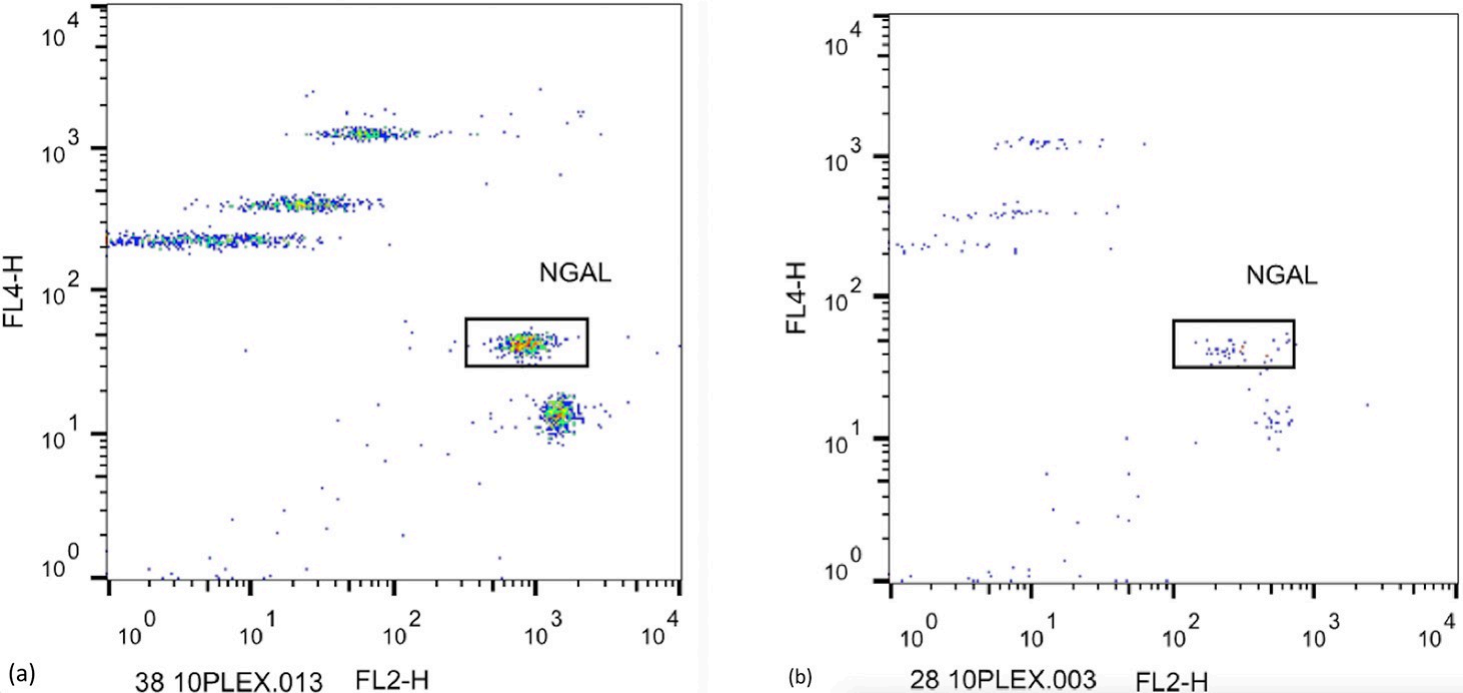
Examples from the expression of NGAL in two cases. Each dot-plot has two parameters. FL2 (horizontal axis) represents the fluorescence intensity related to the concentration of measured factors such as NGAL (framed in a box) and the FL4 (vertical axis) represents the fluorescent intensity by which all measured factors in the same kit are distinguished, allocating them on different sites in the plot.

Fig 2 illustrates vitreous expression of LCN2 (NGAL) in a RRD (a) and a (FTMH) (b), respectively.

### Statistical analysis

Quantitative variables did not follow the normal distribution and were summarized as median (interquartile range), whereas categorical data were expressed as percentages (frequencies) of the groups. Non-normally distributed data were compared using nonparametric methods to assess statistical significance. For quantitative variables, differences between two groups were evaluated with Mann–Whitney U test. Kruskal–Wallis test was applied in cases of more than two groups of quantitative variables. Correlation between two quantitative variables was quantified using Spearman’s correlation coefficient. Variability between the RRD and the control group in terms of age and gender was assessed with independent-samples t-test and chi-square test. To eliminate confounding, we then performed multivariate linear regression analysis using LCN2 levels in the RRD group as the dependent variable. Since the latter did not follow a normal distribution pattern, we used its logarithm for further analysis. This was followed by a backward stepwise elimination method which enabled us to calculate coefficient beta, 95% confidence interval and p-values. Sensitivity analysis was performed in case of presence of outliers. A two tailed p-value of less than 0.05 was considered statistically significant. Statistical analysis was performed with the Statistical Package for Social Sciences software (IBM Corp. Released 2012. IBM SPSS Statistics for Windows, Version 21.0. Armonk, NY: IBM Corp.).

## Results

### Demographics

There were no differences in age and gender between RRD group and controls. In particular, mean age in the RRD group was 64.1 years (standard deviation [SD] = 13.2) while in the control group was 69.9 years (SD = 9.1) (t = -1.2, p = 0.2). In RRD patients 85% (n = 17) were males, while in controls it was 50% (n = 7) were males (x^2^ = 4.9, p = 0.054).

**Fig 3:**
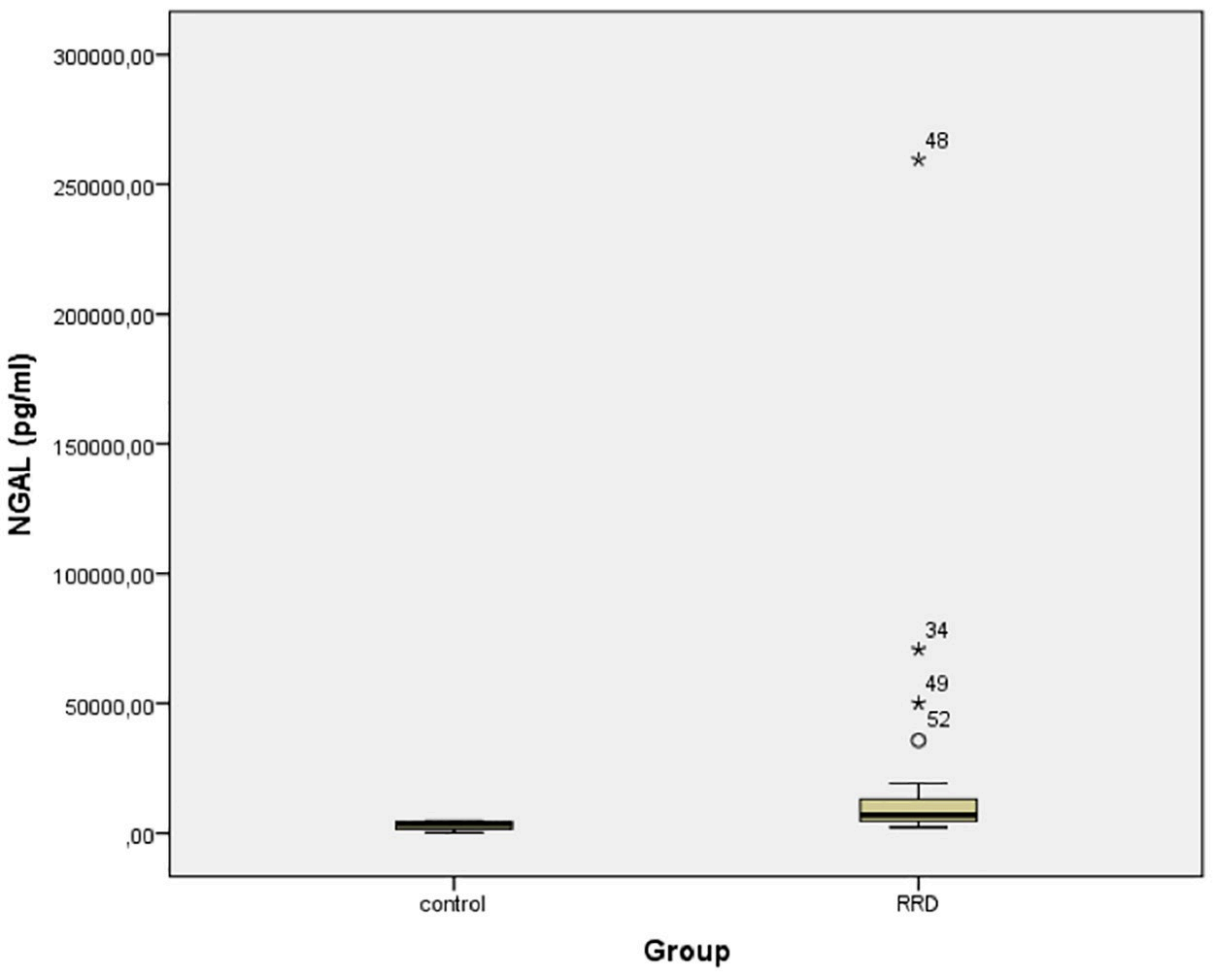
LCN2 (NGAL) levels of the patients and the controls (U = 27, p<0.001). Circles denote mild outliers and asterisks denote extreme outliers.

LCN2 levels of the patients and the controls are shown in Table 1. The median concentration of LCN2 was markedly higher in patients with RRD than in controls (U = 27, p<0.001). In particular, median LCN2 concentration in the RRD group was 7,095 pg/ml (IR = 9,605), while in controls was 3,289 pg/ml (IR = 3,090) (Fig 3).

**Table 1:** LCN2 levels of patients and controls.

	LCN2 levels							P-value	P-value after sensitivity analysis
	Mean	Standard deviation	Median	Minimum value	Maximum Value	Interquartile range	N		
**Controls**	2,975	1,634	3,289	200	4,803	3,090	**10**	**<0.001**^a^	**0.001**^a^
**RRD (total)**	23,481	52,926	7,095	2,311	259,625	9,605	**24**		
**Lens status**								**0.007**^a^	**0.082**^a^
Phakic	5,824	2,720	4,879	2,311	10,527	4,550	**12**		
Pseudophakic	41,137	71,899	13,124	2,520	259,625	40,014	**12**		
**PVR grading**								**<0.001**^b^	**<0.001**^b^
No PVR	3,779	1,209	3,921	2,311	5,561	2,132	**7**		
PVR A	5,084	358	4,916	4,541	5,495	Non computable	**3**		
PVR B	8,609	1,879	9,387	6,055	10,763	3,721	**7**		
PVR C	65,938	88,032	35,759	10,527	259,625	55,346	**7**		
**Quadrants**								**<0.001**^a^	**<0.001**^a^
1–2	3,779	1,209	3,921	2,311	5,561	2,132	**7**		
3–4	31,593	61,534	10,005	4841	259,625	21,316	**17**		
**Symptoms duration (days)**								**<0.001**^b^	**<0.001**^b^
<10	4,023	1,155	4,479	2,311	5,561	2,113	9		
11–30	15,580	19,802	9625	5495	70,883	6,649	10		
>30	74,304	104,81	35,759	10,527	259,625	141,867	5		

^a^ Mann-Whitney

^b^ Spearman’s correlation coefficient

We found a positive correlation between LCN2 concentration and PVR grading (r_s_ = 0.94, p<0.001). Median concentration of LCN2 in PVR C group was 35,759 pg/ml (IR = 55,347), in PVR B group was 9,387 pg/ml (IR = 3,721), in PVR A group was 4,916 pg/ml (IR = non computable) and in no PVR group was 3,921 pg/ml (2,132).

Regarding the lens status in the RRD group, the median LCN2 concentration was significantly higher in pseudophakic compared to phakic patients (U = 25, p = 0.007). In particular, median LCN2 concentration in pseudophakic patients was 13,124 pg/ml (IR = 40,014), while in phakic patients was 4,879 pg/ml (IR = 4550).

The extent of retinal detachment seems to be correlated with the levels of LCN2 (U = 3, p<0.001). In particular, median LCN2 concentration in patients with 3–4 quadrants of involvement was 10,005 pg/ml (IR = 21,316), while in patients with 1–2 quadrants of involvement was 3,921 pg/ml (IR = 2132).

There was also a positive correlation between symptom duration and LCN2 concentration in the RRD group (r_s_ = 0.87, p<0.001). In RRD patients, symptomatic for more than 30 days, median LCN2 was 35,759 pg/ml (IR = 141,867). In patients with symptom duration of 10 to 30 days the median LCN2 concentration was 9,625 pg/ml (IR = 6,649). In RRD patients having symptoms less than 10 days, the median concentration of LCN2 was 4,479 pg/ml (2113) (x^2^ = 17.7, p<0.001).

After multivariate linear regression analysis, only PVR was independently correlated with LCN2 concentration. In particular, increased PVR grading was associated with increased LCN2 concentration (coefficient b = 2.97, 95% confidence interval = 1.89 to 4.67, p<0.001, R^2^ = 66%). Results did not change after sensitivity analysis (coefficient b = 1.57, 95% confidence interval = 1.41 to 1.76, p<0.001, R^2^ = 79%).

## Discussion

In this study, we found elevated LCN2 levels in the vitreous of RRD patients. To our knowledge, this has not been reported to the literature so far. Lipocalin-2 is regarded as a biomarker for several inflammatory diseases [32]. Recent studies have revealed its regulatory role in ocular inflammation and retinal degeneration observed in the setting of diseases such as Stargardt, retinitis pigmentosa and age related macular degeneration [37]. *Tanu Parmar et al*, also described that LCN2 contributes to acute stress response and immune activation of Retinal Pigmented Epithelium (PRE) cells in Abca4^-/-^Rdh8^-/-^ mice after light exposure [38]. Moreover, *Mallika Vapalla et*. *al* correlated the increase in RPE Lipocalin-2 levels with chronic inflammatory conditions, in Cryba1 cKO mice simulating age-related macular degeneration (AMD) [39].

LCN2 is also known to modulate matrix metalloproteinase-9 (MMP-9) activity [40–43]; a mediator involved in normal, as well as in the pathologic would healing characterizing PVR [44–46]. LCN2 seems to play a role in reactive gliosis and neuroinflammation [47, 48], components which are also implicated in PVR pathophysiology [7, 49, 50].

Limitations of this study include the number of recruited patients and the unknown time interval between phacoemulsification and retinal detachment when comparing phakic and pseudophakic patients with RRD.

In summary, we found elevated vitreous levels of LCN2 in patients with RRD and an association between LCN2 expression and PVR grade. Moreover, the vitreous LCN2 overexpression seems to be correlated with some other relevant clinical parameters such as the lens status, the extent of the detachment as well as the symptom duration. Our findings highlight the potential role of lipocalin2 in the development and progression of PVR, supplementing previous studies trying to elucidate its role in inflammation and reactive gliosis. Larger prospective studies are highly encouraged in order to determine the precise role of lipocalin2 as a potential PVR biomarker or therapeutic target.

## Supporting information

S1 Datasheet Summary table of the data collected from each patient record. (DOCX)
