# Supplementary material for: Vitreous levels of Lipocalin-2 on patients with primary rhegmatogenous retinal detachment
Source: PLoS One. 2019 Dec 31;14(12):e0227266. doi: 10.1371/journal.pone.0227266 (PMC6938320; doi:10.1371/journal.pone.0227266)
Supplement: S1 Datasheet — (DOCX) [file pone.0227266.s001.docx]

**Summary table of the data collected from each patient record**

| SAMPLE | 10PLEX | AGE | GENDER | DISEASE | LENS | QUADRANTS | DURATION | PVR | NGAL |
| --- | --- | --- | --- | --- | --- | --- | --- | --- | --- |
| 44 | 5 | 84 | Female | VMT | N/A | N/A | N/A | N/A | 2743.50 |
| 53 | 44 | 72 | Male | VMT | N/A | N/A | N/A | N/A | 200.00 |
| 22 | 26 | 62 | Female | ERM | N/A | N/A | N/A | N/A | 4803.80 |
| 25 | 27 | 74 | Female | ERM | N/A | N/A | N/A | N/A | 4518.70 |
| 5 | 1 | 63 | Female | ERM | N/A | N/A | N/A | N/A | 2986.20 |
| 6 | 2 | 72 | Female | ERM | N/A | N/A | N/A | N/A | 3592.60 |
| 49 | 54 | 73 | Male | ERM | N/A | N/A | N/A | N/A | 4576.60 |
| 54 | 56 | 77 | Male | ERM | N/A | N/A | N/A | N/A | 1678.00 |
| 7 | 3 | 71 | Female | FTMH | N/A | N/A | N/A | N/A | 3921.80 |
| 8 | 4 | 51 | Female | FTMH | N/A | N/A | N/A | N/A | 735.10 |
| 29 | 31 | 67 | Male | RRD | PHAKIC | 1-2 | <10 days | NO | 5561.50 |
| 43 | 14 | N/A | Female | RRD | PHAKIC | 1-2 | <10 days | NO | 3010.20 |
| 45 | 15 | 77 | Male | RRD | PSEUDOPHAKIC | 1-2 | <10 days | NO | 2520.40 |
| 46 | 16 | 42 | Male | RRD | PHAKIC | 1-2 | <10 days | NO | 2311.10 |
| 47 | 32 | 65 | Male | RRD | PHAKIC | 1-2 | <10 days | NO | 4479.60 |
| 1 | 40 | 63 | Male | RRD | PHAKIC | 1-2 | <10 days | NO | 4653.00 |
| 50 | 41 | 35 | Female | RRD | PHAKIC | 1-2 | <10 days | NO | 3921.80 |
| 9 | 33 | 70 | Male | RRD | PHAKIC | 3-4 | <10 days | A | 4841.30 |
| 15 | 35 | 57 | Male | RRD | PHAKIC | 3-4 | <10 days | A | 4916.50 |
| 40 | 13 | N/A | Male | RRD | PSEUDOPHAKIC | 3-4 | 10-30 days | A | 5495.20 |
| 32 | 7 | 59 | Female | RRD | PSEUDOPHAKIC | 3-4 | 10-30 days | B | 10763.20 |
| 36 | 10 | 53 | Female | RRD | PSEUDOPHAKIC | 3-4 | 10-30 days | B | 7907.90 |
| 37 | 11 | 61 | Male | RRD | PHAKIC | 3-4 | 10-30 days | B | 6283.90 |
| 39 | 12 | 61 | Female | RRD | PSEUDOPHAKIC | 3-4 | 10-30 days | B | 9862.80 |
| 13 | 34 | 52 | Male | RRD | PSEUDOPHAKIC | 3-4 | 10-30 days | B | 6055.10 |
| 16 | 36 | 81 | Female | RRD | PHAKIC | 3-4 | 10-30 days | B | 10005.20 |
| 17 | 37 | 48 | Female | RRD | PHAKIC | 3-4 | 10-30 days | B | 9387.36 |
| 33 | 8 | 62 | Male | RRD | PSEUDOPHAKIC | 3-4 | 10-30 days | C | 19213.70 |
| 35 | 9 | 64 | Male | RRD | PSEUDOPHAKIC | 3-4 | 10-30 days | C | 70833.50 |
| 18 | 38 | 86 | Male | RRD | PSEUDOPHAKIC | 3-4 | >30 days | C | 259625.10 |
| 2 | 39 | 82 | Male | RRD | PSEUDOPHAKIC | 3-4 | >30 days | C | 50124.00 |
| 52 | 42 | 80 | Male | RRD | PSEUDOPHAKIC | 3-4 | >30 days | C | 35759.00 |
| 57 | 43 | 72 | Male | RRD | PSEUDOPHAKIC | 3-4 | >30 days | C | 15486.60 |
| 50 | 28 | 74 | Male | RRD | PHAKIC | 3-4 | >30 days | C | 10527.60 |
